# The fatty acid profile of adipose tissue as a predictor of the ponderal and inflammatory response in adult women six years after bariatric surgery

**DOI:** 10.1186/s12944-020-01229-3

**Published:** 2020-03-16

**Authors:** Crislaine das Graças de Almeida, Elaine Cristina Viana, Ana Vládia Bandeira Moreira, Gustavo Peixoto Soares Miguel, Fernanda Semião Garcia Pedra, Fabiana Eleotério Oliveira, Tayla Neves Quimquim, Nazaré Souza Bissoli, Raquel Duarte Moreira Alves, Josefina Bressan

**Affiliations:** 1 Department of Nutrition and Health, Federal University of Viçosa, Viçosa, Minas Gerais Brazil; 2 Campus Boa Vista, Vila Velha University, Vila Velha, Espírito Santo Brazil; 3 Biological Sciences Institute, Federal University of Juiz de Fora, Juiz de Fora, Minas Gerais Brazil; 4 Department of Clinical Surgery, Federal University of Espírito Santo, Vitória, Espírito Santo Brazil; 5 Santa Rita de Cássia Hospital, Vila Velha University, Vila Velha, Espírito Santo Brazil; 6 Biomedical Center, Department of Physiological Sciences, Federal University of Espírito Santo, Vitória, Espírito Santo Brazil

**Keywords:** Fatty acid, IL-6, TNF, Weight loss, Roux-en-Y gastric bypass, Sleeve gastrectomy

## Abstract

**Background:**

Adipose tissue is involved in several metabolic changes. This study investigated the association between the fatty acid (FA) composition of subcutaneous (SAT) and visceral (VAT) adipose tissue pre-surgery and the postsurgical response regarding the evolution of weight and concentrations of tumour necrosis factor alpha (TNF) and interleukin 6 (IL-6) in adult women who underwent Roux-en-Y gastric bypass (RYGB, *n* = 14) or sleeve gastrectomy (SG, *n* = 19) at one (T1), three (T3) and six (T6) years after surgery.

**Methods:**

Blood samples were collected to obtain plasma for the measurement of IL-6 and TNF. Anthropometric measurements were performed, collecting samples of VAT and SAT during surgery to assess the FA profiles.

**Results:**

Weight loss had a positive correlation with the percentage of VAT-C17:0 (T1, T3) and SAT-C18:2 (T1, T3, T6), and it had a negative correlation with SAT-C22:0 (T1, T3) and VAT-C22:0 (T3). Regarding the inflammatory response, SAT-C14:0 (T6), VAT-C14:0 (T6), SAT-C14:1 (baseline), SAT-C15:0 (T6), SAT-C16:1 (T6), VAT-C16:1 (baseline), SAT-C17:1 (T6), VAT-C17:1 (baseline), VAT-C18:1 (T6), and VAT-C20:1 (T6) exhibited positive correlations with the concentration of IL-6, which were different from the correlations of IL-6 concentrations with SAT-C18:2, VAT-C18:2 (T6), and VAT-C18:3 (T6). The FA SAT-C18:0 (T1) was negatively correlated with TNF concentrations.

**Conclusions:**

Saturated FAs were predominantly proinflammatory, primarily in the late postoperative period. Alternately, the polyunsaturated FAs exhibited anti-inflammatory potential and predicted weight loss. Thus, the FA profile of the adipose tissue of obese adult women may be a predictor of the ponderal and inflammatory response 6 years after bariatric surgery.

**Trial registration:**

This study was approved by the ethics committee of Federal University of Viçosa; Registration n. 17287913.2.0000.5153; Date: 07/05/2013.

## Background

Adipose tissue (AT) has been associated with different metabolic disorders [1] and is directly involved in the genesis of obesity [2]. The location of AT interferes with the metabolic response, as visceral adipose tissue (VAT) is more active, insulin-resistant, and lipolytic, favoring the release of free fatty acids (FAs) in the blood [3], while subcutaneous adipose tissue (SAT) is more willing to receive FAs and triacylglycerols from circulation. Additionally, VAT is more vascularized and innervated and contain more immune and inflammatory cells [4].

FAs exert vital functions in humans as structural and functional constituents of the cellular membrane [5], and they are bioactive lipids for the synthesis of ATP, resulting from the catabolism of triacylglycerols [6]. Furthermore, the essential FAs of the omega-3 and omega-6 series, consumed in adequate proportions, are beneficial, acting on inflammatory pathways and protecting against various pathologies, such as cardiovascular disease, type 2 diabetes, and dyslipidemia [7]. However, the proportion of saturated and trans FAs in AT may be associated with an increased risk for the development of chronic noncommunicable diseases [8, 9] and may influence the ability of AT to secrete growth factors [10], hormones, and adipokines [11].

Among the proinflammatory adipokines, tumour necrosis factor alpha (TNF) and interleukin 6 (IL-6) are highlighted, primarily because they have concentrations that are positively correlated with the amount of body fat and body mass index (BMI) [12, 13] and negatively correlated with weight loss (WL), either by dietary restriction and physical activity [14] or by surgical intervention [15, 16]. According to the American Society for Metabolic and Bariatric Surgery (ASMBS) [17], bariatric surgery is indicated as the most effective and long-lasting treatment to achieve a healthy nutritional state, with attenuation or the eradication of associated disorders, including type 2 diabetes, hypertension, hypercholesterolemia, nonalcoholic hepatic steatosis, and obstructive sleep apnea.

AT is an endocrine organ [18] capable of significantly influencing the metabolism of severely obese individuals. These individuals may absorb more FA at the intestinal level than healthy and nonobese individuals [19], and the type of FA may influence metabolic repercussions and weight loss [20]. Thus, the present study aims to investigate the association between the composition of FA from preoperative VAT and abdominal SAT and the postsurgical response regarding the evolution of weight as well as TNF and IL-6 concentrations in women submitted to two different bariatric techniques during the presurgery (baseline) period and at 1, 3, and 6 years postsurgery.

## Materials and methods

### Participants

Initially, 65 women participating in the Obesity and Bariatric Surgery Program of the Federal University of Espírito Santo (UFES) were selected for the project “Results of bariatric and metabolic surgery: vertical gastrectomy versus vertical gastroplasty with Y-de-roux shunt. Prospective clinical trial,” conducted by the working group [21, 22]. The selected patients met the inclusion criteria for bariatric surgery [17, 21], being identified among candidates for bariatric surgery seeking the Program; a greater prevalence of women between 20 and 60 years and with BMI between 40 and 45 kg/m^2^ were selected for the study group for a greater homogeneity of the sample. Respecting the ethical standards of the selection process, each woman chose between the two types of surgery to be performed by the team after clarification on the procedures. Of the 65 women, 33 underwent up to 6 years of follow-up; 14 underwent Roux-en-Y gastric bypass (RYGB) and 19 underwent sleeve gastrectomy (SG), with collections of blood samples at baseline (T0) and at one (T1), three (T3), and six (T6) years postsurgery. All women were enrolled in the UFES Obesity and Bariatric Surgery Program and followed clinically by all team members in parallel with this work.

### Anthropometry, body composition, and derivative measures

Weight, height [23], waist circumference (WC), and hip circumference (HC) [24] were measured. The BMI and the waist-to-hip ratio (WHR) were calculated [25]. Bioelectrical impedance analysis was performed using a multifrequency impedance analyzer (Multiscan5000, Bodystat Ltd., Isle of Man, UK) and it was used to evaluate body fat percentage (BF%), lean body mass percentage (LM%), and basal metabolic rate (BMR) [26].

To analyze the impact of the intervention on weight loss with respect to time after surgery, the following indicators were used: absolute weight loss (WL), percentage of weight loss (%WL), and percentage of excess BMI lost (%EBMIL) [27, 28], according to the equations: WL = initial weight (kg) - postsurgery weight (kg); %WL = [WL (kg)/initial weight (kg)] × 100; and %EBMIL = [initial BMI - BMI after surgery (kg/m^2^)] × 100/[initial BMI - ideal BMI (kg/m^2^)].

### Measurement of proinflammatory cytokines

Plasma levels of the proinflammatory cytokines IL-6 and TNF among the 33 participants were collected at the T0, T1, T3, and T6 timepoints. The blood samples were collected after 10 h of fasting and frozen in liquid nitrogen after collection. The concentrations were determined by the MILLIPLEX MAP High Sensitivity Human Cytokine Panel method [29]. The sensitivity estimates of this method for the IL-6 and TNF assays were 0.10 pg/ml and 0.05 pg/ml, respectively. The accuracy was 93–112%, and the standard curve range was 0.13–2000 pg/ml. The detection method used was Luminex xMAP.

### Analysis of the FA profile of AT

The FA profile of the VAT and SAT was determined by gas chromatography (GC) at the Food Analysis Laboratory of the Department of Nutrition and Health of the Federal University of Viçosa and at the Laboratory of Biochemical Analysis in Institute of Biotechnology Applied to Agriculture (BIOAGRO) of the Federal University of Viçosa.

The total FAs of the tissues were extracted according to the Folch method [30] and esterified by the Hartman and Lago method [31]. For this purpose, during bariatric surgery, VAT and SAT samples were collected, frozen in liquid nitrogen and stored at − 80 °C. For analysis, 35 mg tissue samples were used in duplicate. To this 35 mg aliquot, 1.9 ml chloroform:methanol reagent (2:1) was added, followed by glass rod maceration and vortex mixing for 3 min. To this mixture, 0.4 ml of methanol was added, and the mixture was centrifuged for 10 min at 3000 rpm. The supernatant was then transferred to a capped tube, and 0.8 ml of chloroform and 0.64 ml of 0.73% NaCl were added. After being vortexed again for 1 min, the capped tube was centrifuged for an additional 10 min at 3000 rpm. The upper phase was discarded, and the tube wall was washed with 0.3 ml Folch solution; this process was repeated 3 times. As the last step of the process, the uncapped tubes were left in a semi-open oven at 37 °C until the following day.

After extraction, the lipids were esterified by the Hartman and Lago technique [31]. To the test tube containing the dried extract, 4 ml of saponification reagent (2% NaOH in methanol) was added, and the tubes were left in a water bath at 80 °C for 15 min. Then, 3 ml of the esterification reagent (2 g of ammonium chloride + 60 ml of methanol + 3 ml of concentrated sulfuric acid) was added to the same tube. This tube was again placed in a water bath at 80 °C for 15 min and then cooled to approximately 40 °C. After cooling, 1.5 ml of 20% sodium chloride and 0.5 ml of hexane (HPLC grade) were added and subsequently vortexed. The supernatant was transferred to labeled Eppendorf tubes. To the remainder, 0.5 ml of hexane was added, and the supernatant was transferred to the same Eppendorf tube. The Eppendorf tube contents were dried in nitrogen and frozen in a − 20 °C freezer, protected from light and humidity, until read on the gas chromatograph.

For identification of the fatty acid methyl esters (FAME), a GC-20 Shimadzu (Kyoto, Japan) chromatograph equipped with an FID detector was used. For recording and analysis of the chromatograms, the device was coupled to a microcomputer using the GC Solution program. The compounds were separated on a Carbowax capillary column (100 m × 0.25 mm) and identified by comparison to the relative retention times of FAME peaks from samples, with those of the standard mixture Supelco® 37 component FAME Mix C4:0-C24:0 (Sigma-Aldrich; St. Louis, MO, USA) and the results were expressed as percentage of the area.

The chromatographic analysis required 1 μL of sample, which was injected with a 10 μL syringe (Hamilton®, Sigma-Aldrich; St. Louis, MO, USA) into a Split = 10 system. For analytical gases, nitrogen gas was used as the carrier, with a linear velocity programmed to 27.3 cm/s, and hydrogen and synthetic air gases formed the detector flame. Injector and detector temperatures were controlled isothermally at 200 °C and 220 °C. For chromatographic separation, the following temperature programming was used: the initial column temperature was 40 °C (maintained for 2 min), then it was increased by 4 °C per minute until reaching 220 °C (maintained for 30 min). The carrier gas flow in the column was 0.8 ml/min.

### Statistical analyses

The results are presented as the median (interquartile range - IQR) and mean ± standard deviation (SD) and were analyzed using SAS Software Version 9.0 (SAS Institute Inc. 2000). To evaluate the data regarding the comparison between the study periods, repeated measurements ANOVA was used for the parametric data, and the Friedman test was used for nonparametric data. If there was a difference between the times analyzed, the appropriate post hoc test was applied (Tukey-Kramer or Dunn).

The Kruskal-Wallis test was used to assess the proportions of each FA in each tissue, followed by the Dunn test. For the comparison between the study groups (RYGB and SG), Student’s t-test was used for the variables that presented a normal distribution, and the Mann-Whitney test was used for variables that did not present a normal distribution. To analyze the association between the variables, the Spearman correlation test and multiple linear regression were used. The level of significance adopted was *p* < 0.05.

## Results

### Anthropometric variables and body composition

There was no significant difference between the RYGB and SG groups at all collection times (Supplemental Table 1) with regard to age, weight, height, BMI, WC, HC, BF% and MM%. Except for WHR at baseline and T1, all anthropometric and body composition variables at baseline were different (*p* < 0.0001) from the other collection times (T1, T3, and T6), both when considering the total number of patients and when analyzing by surgery group (Supplemental Table 2).

The weight and BMI values presented the same behavior for both surgical techniques, and there was no difference (*p* > 0.05) between T1 and T3. Concerning the classification of BMI, it was observed that at T1, T3, and T6, the patients were overweight. However, there was an increase in body weight and, therefore, BMI at T6 (*p* < 0.05). Additionally, BMR was affected by the type of surgery (*p* = 0.0045) and by the collection time (*p* < 0.0001), with higher values in the RYGB group at T1 (*p* = 0.0241) and T6 (*p* = 0.0296) (Supplemental Table 2).

Regarding the relative anthropometric variables studied, namely, WL (kg), %WL, and %EBMIL, it is noteworthy that WL (kg) was influenced by both the type of surgery and the collection time (*p* < 0.0001), and there was an interaction between the type of surgery and the collection time (*p* < 0.0001). On the other hand, %WL was not affected by the type of surgery, but it was affected by the collection time (*p* < 0.0001). However, it presented a group versus time interaction (*p* = 0.0274). Although %EBMIL had been influenced by collection time (*p* < 0.0001), it was not influenced by the type of surgery. Moreover, there was no observed interaction between the type of surgery and time (*p* > 0.05) (Table 1).

**Table 1:** Anthropometric variables related to the baseline at collection times

Variable	1 year’ surgery			3 years’ surgery			6 years’ surgery		
	SG + RYGB	SG	RYGB	SG + RYGB	SG	RYGB	SG + RYGB	SG	RYGB
**WL (kg)**	37.6 (32.7–45.6) a (38.3 ± 7.1)	36.0 (33.8–41.6) (37.5 ± 6.1)	39.9 (31.1–46.3) b (39.4 ± 8.5)	37.9 (31.0–43.5) b (37.7 ± 9.3)	36.9 (30.2–41.3) (36.0 ± 8.3)	40.7 (31.9–47.1) a (39.9 ± 10.4)	32.6 (25.0–40.1) c (31.8 ± 10.9)	32.6 (27.6–36.8) (31.6 ± 7.2)	31.8 (19.1–42.0) ac (32.0 ± 14.9)
**%WL**	34.6 (30.4–39.1) b (34.8 ± 5.9)	33.0 (30.6–40.4) ab (35.0 ± 6.4)	35.5 (31.2–37.9) b (34.5 ± 5.4)	39.6 (32.2–49.3)a (41.9 ± 12.5)	38.2 (32.0–43.8)a (38.7 ± 9.0)	46.5a (46.1 ± 15.5)	29.7 (22.5–35.8)c (28.9 ± 9.7)	29.7 (25.1–35.8)b (29.6 ± 7.4)	28.1 (17.6–37.3) b (28.0 ± 12.4)
**%EBMIL**	85.7 (74.1–92.5) a (84.3 ± 14.4)	84.3 (74.6–99.5) a (85.1 ± 15.2)	86.4 (72.5–92.0) a (83.2 ± 13.9)	83.4 (71.4–97.8) b (82.8 ± 20.0)	84.1 (70.3–97.9) ab (81.5 ± 22.5)	81.1 (72.2–94.8) b (84.5 ± 16.8)	76.2 (56.3–84.9) c (69.6 ± 24.5)	76.4 (59.5–84.1) b (70.3 ± 21.4)	75.5 (44.3–95.0) c (68.8 ± 29.1)

Values are expressed as median (interquartile range [IQR]) and mean ± standard deviation. Lowercase letters indicate the difference (*p* < 0.05) among the groups at each collection time (Shapiro-Wilk followed by t test or Mann-Whitney). Upper case letters indicate difference (*p* < 0.05) among collection times for each group (Repeated measures ANOVA, followed by the Tukey-Kramer test)

*SG* sleeve gastrectomy (*n* = 19), *RYGB* Roux-en-Y bypass (*n* = 14), *WL* weight loss, *%WL* weight loss percentage, *%EBMIL* percentage of excess BMI that was lost

### Proinflammatory cytokines: IL-6 and TNF

**Fig. 1:**
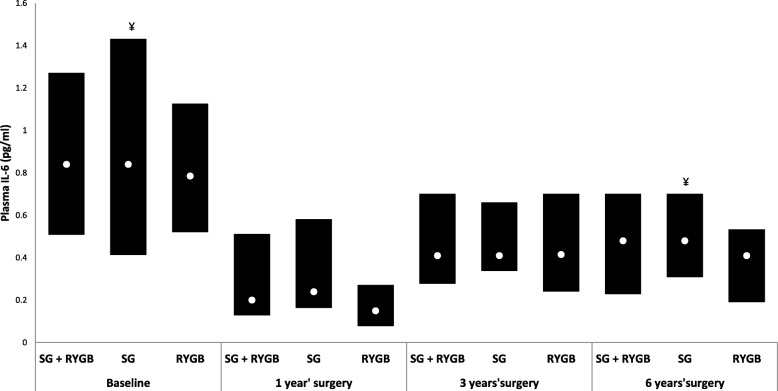
Median and interquartile range of IL-6 (pg/ml) plasma values at baseline and at 1, 3 and 6 years postsurgery among women undergoing two bariatric surgery techniques. Repeated measures ANOVA followed by Tukey-Kramer. The symbol ¥ indicates the difference (*p* = 0.01) of the group at the indicated collection time compared to the other collection times. Bars marked with the same symbol are equal

IL-6 concentrations were equal among the collection times and the types of surgery in each collection, except for the difference between T1 and T6 in the SG group (*p* = 0.0212), with lower values at the latter time (Fig. 1).

**Fig. 2:**
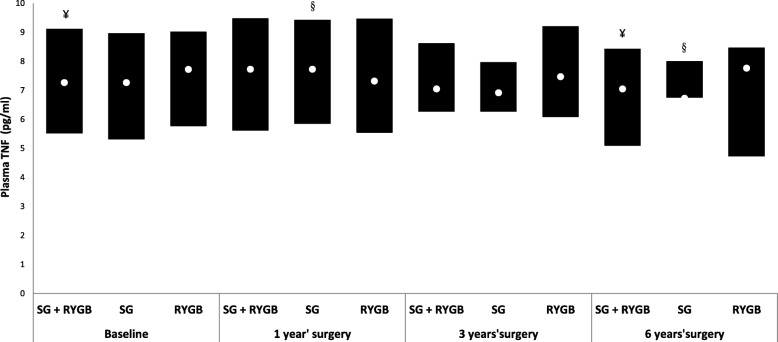
Median and interquartile range of TNF (pg/ml) plasma values at baseline and at 1, 3 and 6 years postsurgery among women undergoing two bariatric surgery techniques. Repeated measures ANOVA followed by Tukey-Kramer. The symbols (¥,§) indicate the difference (¥ *p* = 0.01; § *p* = 0.02) of the group at the indicated collection time compared to the other collection times. Bars marked with the same symbol are equal

TNF was lower at T6 in the total sample (*p* = 0.0156) as well as in the SG group (*p* = 0.0156). Regarding the RYGB group, no difference was observed (*p* > 0.05). There was no difference in the TNF levels among the types of surgery at any of the collection times (Fig. 2).

### Composition of FA from VAT and SAT before bariatric surgery

Concerning SAT, among other observations, a higher proportion of C18:1 was found, followed by C18:2, C16:0, C18:0 and C16:1 (Table 2). However, for VAT, greater proportions of C18:1 were observed, followed by C18:2, C16:0, C16:1, and C18:0. The remaining FAs did not differ (Table 2). When comparing tissues, higher values of C14:1 (*p* = 0.0097), C16:1 (*p* = 0.0011) and C20:1 (*p* = 0.0046) were observed in VAT, while higher concentrations of FAs C16:0 (*p* = 0.0446), C17:0 (*p* = 0.0239), C20:2 (*p* = 0.0009), C22:0 (*p* < 0.0001), and C20:4 (*p* < 0.0001) were observed in SAT (Table 2).

**Table 2:** Fatty acid profile of the SAT and VAT lipid fractions (%)

FA (%)	SAT			VAT		
	SG + RYGB	SG	RYGB	SG + RYGB	SG	RYGB
**C12:0**	0.08 (0.04–0.11) (0.09 ± 0.06)	0.08 (0.06–0.11) (0.09 ± 0.05)	0.06 (0.04–0.11) (0.09 ± 0.07)	0.09 (0.07–0.11) (0.09 ± 0.04)	0.09 (0.06–0.11) (0.09 ± 0.05)	0.09 (0.08–0.10) (0.09 ± 0.02)
**C14:0**	1.17 (0.99–1.53) (1.23 ± 0.30)	1.16 (0.95–1.43) (1.22 ± 0.33)	1.19 (0.99–1.53) (1.25 ± 0.26)	1.24 (1.02–1.54) (1.28 ± 0.42)	1.16 (1.02–1.51) (1.27 ± 0.49)	1.32 (1.06–1.57) (1.28 ± 0.34)
**C14:1**	0.11 (0.09–0.13) b (0.12 ± 0.05)	0.11 (0.09–0.13) (0.12 ± 0.06)	0.12 (0.08–0.13) (0.11 ± 0.04)	0.18 (0.11–0.20) a (0.16 ± 0.06)	0.18 (0.12–0.20) (0.16 ± 0.06)	0.18 (0.10–0.20) (0.16 ± 0.06)
**C15:0**	0.18 (0.15–0.21) (0.18 ± 0.04)	0.18 (0.14–0.22) (0.18 + 0.04)	0.18 (0.15–0.21) (0.18 ± 0.04)	0.17 (0.13–0.20) (0.17 ± 0.04)	0.17 (0.13–0.22) (0.17 ± 0.04)	0.17 (0.16–0.18) (0.17 ± 0.04)
**C16:0**	20.88 (19.97–23.05) a (21.25 ± 2.48)	20.51 (19.79–22.38) (20.83 ± 2.28)	21.60 (20.64–23.48) (21.80 ± 2.73)	20.05 (19.12–21.29) b (20.14 ± 1.86)	19.88 (19.16–20.13) (19.73 ± 1.61)	21.04 (19.15–22.19) (20.70 ± 2.08)
**C16:1**	3.47 (2.94–4.33) b (3.55 ± 1.18)	3.86 (3.00–4.52) (3.73 ± 1.37)	3.14 (2.80–3.50) (3.29 ± 0.83)	4.36 (3.78–5.42) a (4.67 ± 1.46)	4.84 (3.60–5.46) (4.72 ± 1.43)	4.28 (3.85–4.78) (4.60 ± 1.56)
**C17:0**	0.21 (0.20–0.26) a (0.31 ± 0.43)	0.20 (0.19–0.24) (0.22 ± 0.05)	0.22 (0.21–0.27) (0.43 ± 0.67)	0.19 (0.16–0.22) b (0.20 ± 0.05)	0.21 (0.17–0.22) (0.20 ± 0.03)	0.17 (0.15–0.22) (0.19 ± 0.06)
**C17:1**	0.25 (0.22–0.27) (0.24 ± 0.04)	0.26 (0.23–0.27) (0.25 ± 0.04)	0.22 (0.20–0.27) (0.23 ± 0.04)	0.26 (0.24–0.31) (0.27 ± 0.06)	0.26 (0.25–0.31) (0.28 ± 0.06)	0.25 (0.23–0.29) (0.26 ± 0.05)
**C18:0**	3.22 (2.70–4.19) (3.70 ± 1.68)	3.12 (2.74–3.74) (3.44 ± 1.49)	3.51 (2.64–4.91) (4.04 ± 1.89)	3.20 (2.61–3.72) (3.56 ± 1.56)	3.32 (2.57–4.03) (3.72 ± 1.75)	3.15 (2.66–3.32) (3.35 ± 1.31)
**C18:1**	42.36 (40.89–43.76) (41.84 ± 4.52)	42.54 (41.43–43.35) (41.56 ± 5.56)	42.01 (40.82–43.92) (42.21 ± 2.72)	43.62 (41.64–45,20 (42.96 ± 5.57)	43.64 (40.82–45.60) (442.68 ± 7.19)	43.00 (42.02–45.05) (43.34 ± 2.08)
**C18:2**	22.19 (19.93–24.03) (22.05 ± 3.20)	22.19 (20.02–24.07) (22.21 ± 3.01)	22.49 (19.96–23.77) (21.83 ± 3.54)	21.42 (19.65–24.10) (22.04 ± 3.68)	22.66 (20.03–25.16) (22.50 ± 4.33)	20.62 (19.66–22.36) (21.42 ± 2.59)
**C20:0**	0.06 (0.04–0.09) (0.32 ± 1.18)	0.07 (0.05–0.09) (0.07 ± 0.02)	0.05 (0.03–0.10) (0.66 ± 1.81)	0.06 (0.05–0.08) (1.00 ± 4.33)	0.07 (0.06–0.08) (1.59 ± 5.50)	0.05 (0.04–0.06) (0.05 ± 0.03)
**C18:3**	0.89 (0.70–1.03) (0.85 ± 0.24)	0.91 (0.68–1.02) (0.83 ± 0.26)	0.86 (0.75–1.05) (0.86 ± 0.21)	0.89 (0.74–0.99) (0.85 ± 0.24)	0.86 (0.70–1.06) (0.85 ± 0.24)	0.86 (0.76–0.97) (0.82 ± 0.16)
**C20:1**	0.05 (0.04–0.06) b (0.05 ± 0.02)	0.05 (0.04–0.06) (0.52 ± 0.02)	0.05 (0.04–0.05) (0.05 ± 0.01)	0.07 (0.06–0.09) a (0.07 ± 0.02)	0.08 (0.07–0.10) (0.08 ± 0.02)	0.06 (0.05–0.06) (0.06 ± 0.02)
**C21:0**	0.35 (0.32–0.41) (0.37 ± 0.08)	0.34 (0.31–0.40) (0.35 ± 0.07)	0.38 (0.33–0.43) (0.39 ± 0.08)	0.40 (0.31–0.47) (0.39 ± 0.11)	0.41 (0.32–0.46) (0.39 ± 0.12)	0.38 (0.31–0.49) (0.40 ± 0.10)
**C20:2**	0.27 (0.26–0.31) a (0.28 ± 0.05)	0.27 (0.26–0.30) (0.28 + 0.04)	0.29 (0.27–0.32) (0.29 ± 0.05)	0.22 (0.19–0.28) b (0.23 ± 0.06)	0.22 (0.19–0.29) (0.23 ± 0.06)	0.23 (0.21–0.27) (0.24 ± 0.05)
**C22:0**	0.38 (0.31–0.44) a (0.68 ± 1.58)	0.38 (0.30–0.42) (0.36 ± 0.09)	0.38 (0.32–0.63) (1.14 ± 2.46)	0.18 (0.13–0.21)b (0.18 ± 0.06)	0.17 (0.13–0.21) (0.18 ± 0.07)	0.18 (0.13–0.20) (0.17 ± 0.06)
**C20:4**	0.53 (0.49–0.60) a (0.54 ± 0.10)	0.56 (0.52–0.63) a (0.58 ± 0.10)	0.51 (0.42–0.53)B (0.48 ± 0.09)	0.34 (0.25–0.43) b (0.36 ± 0.13)	0.38 (0.30–0.42) (0.37 ± 0.12)	0.26 (0.24–0.41) (0.34 ± 0.12)

Values are expressed as median (interquartile range [IQR]) and mean ± standard deviation. Lowercase letters indicate the difference (*p* < 0.05) among the adipose tissue types (Shapiro-Wilk followed by t test or Mann-Whitney). Upper case letters indicate difference (*p* < 0.05) among the groups, by comparison between surgical techniques, in the same type of adipose tissue (Repeated measures ANOVA, followed by the Tukey-Kramer test for parametric data; or Friedman test followed by the Dunn test for non-parametric data)

*FA* fatty acid, *VAT* visceral adipose tissue, *SAT* subcutaneous adipose tissue, *SG* sleeve gastrectomy (*n* = 19), *RYGB* Roux-en-Y bypass (*n* = 14)

When comparing the surgery groups, for each type of AT, higher values of FA C20:4 (*p* = 0.0134) in the SAT were observed for the SG group. No differences in the VAT FAs were found between the surgery groups (Table 2).

### Associations of tissue FAs with weight loss and proinflammatory cytokines

Baseline IL-6 was positively correlated, though moderately, with C14:1 concentrations of the SAT (*r* = 0.42, *p* = 0.03) (Table 3) and with C16:1 (*r* = 0.40, *p* = 0.02) and C17:1 (*r* = 0.42, *p* = 0.02) FAs of the VAT (Table 4). Additionally, the IL-6 values of T3 showed a moderate positive correlation with the C14:1 concentration of the SAT (*r* = 0.38, *p* = 0.03).

**Table 3:** Significant correlations between FA concentrations of SAT and the parameters: %WL, %EBMIL, and plasma concentrations of IL-6 and TNF at collection times

FA	Baseline	1 year’ surgery	3 years’ surgery	6 years’ surgery
**C14:0**			**IL-6** *r* = 0.38 *p* = 0.03	
**C14:1**	**IL-6** *r* = 0.41 *p* = 0.03			
**C15:0**				**IL-6** *r* = 0.40 *p* = 0.02
**C16:1**				**IL-6** *r* = 0.36 *p* = 0.04
**C17:1**				**IL-6** *r* = 0.46 *p* = 0.01
**C18:0**		**TNF** *r* = − 0.43 *p* = 0.01		
**C18:2**		**%EBMIL:** *r* = 0.41 *p* = 0.02	**%EBMIL:** *r* = 0.44 *p* = 0.01	**%EBMIL:** *r* = 0.38 *p* = 0.03
		**%WL:** *r* = 0.37 *p* = 0.03		**%WL:** *r* = 0.36 *p* = 0.04
				**IL-6:** *r* = − 0.37 *p* = 0.01
**C22:0**		**%EBMIL:** *r* = − 0.37 *p* = 0.04	**%WL:** *r* = − 0.43 *p* = 0.01	

*FA* fatty acid, *SAT* subcutaneous adipose tissue, *%WL* weight loss percentage, *%EBMIL* percentage of excess BMI that was lost., *IL-6* interleukin 6, *TNF* tumor necrosis factor alpha. Correlation test: Spearman (*p* < 0.05)

**Table 4:** Significant correlations between FA concentrations of VAT and the parameters: %WL, %EBMIL, and plasma concentrations of IL-6 and TNF at collection times

Fatty Acid	Baseline	1 year’ surgery	3 years’ surgery	6 years’ surgery
**C14:0**				**IL-6** *r* = 0.41 *p* = 0.02
**C16:1**	**IL-6** *r* = 0.40 *p* = 0.02			
**C17:0**		**%EBMIL:** *r* = 0.37 *p* = 0.04	**%EBMIL:** *r* = 0.36 *p* = 0.049	
**C17:1**	**IL-6** *r* = 0.42 *p* = 0.02			**IL-6** *r* = 0.50 *p* = 0.003
**C18:0**				**%WL:** *r* = − 0.38 *p* = 0.03
				**%EBMIL:** *r* = −0.40 *p* = 0.02
**C18:1**				**IL-6** *r* = 0.36 *p* = 0.03
**C18:2**				**IL-6:** *r* = −0.40 *p* = 0.02
**C18:3**				**IL-6:** *r* = −0.35 *p* = 0.046
**C20:1**				**IL-6:** *r* = 0.46 *p* = 0.04
**C22:0**			**%WL:** *r* = 0.48 *p* = 0.02	

*%WL* weight loss percentage, *%EBMIL* percentage of excess BMI that was lost, *IL-6* interleukin 6, *TNF* tumor necrosis factor alpha. Correlation test: Spearman (*p* < 0.05)

At T6, a moderate positive correlation of IL-6 was observed for the subcutaneous FAs C15:0 (*r* = 0.40, *p* = 0.02), C16:1 (*r* = 0.36, *p* = 0.04) and C17:1 (*r* = 0.46, *p* = 0.01) (Table 3). Moreover, the same applied for the visceral FAs C14:0 (*r* = 0.41, *p* = 0.02), C17:1 (*r* = 0.50, *p* = 0.01), C18:1 (*r* = 0.36; *p* = 0.04), and C20:1 (*r* = 0.46, *p* = 0.04) (Table 4).

However, in relation to T6, moderate negative correlations were observed between the plasma concentrations of IL-6 and the FA C18:2, both of the SAT (*r* = − 0.37, *p* = 0.01) and of the VAT (*r* = − 0,40, *p* = 0.02) (Tables 3 and 4), as well as the FA C18:3 of the VAT (*r* = − 0.35, *p* = 0.046). Regarding TNF concentrations, only a negative and moderate correlation was observed with SAT-C18: 0 (*r* = − 0.43, *p* = 0.01) (Table 3).

Regarding T6, one last observation is that, by grouping the FAs according to the degree of unsaturation, a moderate positive correlation was found between visceral monounsaturated FA and IL-6 concentration (*p* = 0.03), while polyunsaturated FA of the VAT was negatively correlated (*p* = 0.019) with this variable (Table 5).

**Table 5:** Significant correlations between the groups of FAs by degree of unsaturation (saturated, monounsaturated and polyunsaturated) of VAT and the parameters: %WL, %EBMIL, and plasma concentrations of IL-6 and TNF at collection times

FA group	Baseline	1 year’ surgery	3 years’ surgery	6 years’ surgery
**Saturated FA**		**%WL** *r* = − 0.35 *p* = 0.047		
		**%EBMIL** *r* = − 0.36 *p* = 0.037		
**Monounsaturated FA**				**IL-6** *r* = 0.36 *p* = 0.03
**Polyunsaturated FA**				**IL-6** *r* = − 0.41 *p* = 0.019

*FA* fatty acid, *VAT* visceral adipose tissue, *%WL* weight loss percentage, *%EBMIL* percentage of excess BMI that was lost, *IL-6* interleukin 6, *TNF* tumor necrosis factor alpha. Correlation test: Spearman (*p* < 0.05)

In relation to the weight loss measures, a moderate positive correlation of the %EBMIL with SAT-C18:2 (*r* = 0.41, *p* = 0.02) (Table 3) and VAT-C17:0 (*r* = 0.37, *p* = 0.04) (Table 4) at T1 was observed. However, at T1, a moderate negative correlation was observed between %EBMIL and SAT-C22:0 (*r* = − 0.37; *p* = 0.04) (Table 3).

Additionally, the %EBMIL presented a moderate positive correlation with SAT-C18:2, both at T3 (*r* = 0.44, *p* = 0.01) and at T6 (*r* = 0.38, *p* = 0.02) (Table 3), as well as with VAT-C17:0 (*r* = 0.36, *p* = 0.04) at T3 (Table 4). However, %EBMIL showed a negative correlation with VAT-C18:0 at T6 (*r* = − 0.39, *p* = 0.02) (Table 4).

Similar to %EBMIL, the %WL showed a positive correlation with SAT-C18:2 at T1 (*r* = 0.37, *p* = 0.03) and at T6 (*r* = 0.36, *p* = 0,04) (Table 3) and a negative correlation with VAT-C18:0 at T6 (*r* = − 0.38, *p* = 0.03) (Table 4). Furthermore, the %WL exhibited a moderate negative correlation with SAT-C22:0 (*r* = − 0.44; *p* = 0.01) (Table 3) and a moderate positive correlation with VAT-C22:0 (*r* = 0.48, *p* = 0.02) at T3 (Table 4).

Finally, after grouping by degrees of unsaturation, moderate positive correlations were observed between polyunsaturated FAs and %EBMIL at all collection times with respect to SAT (T1: *p* = 0.04; T3: *p* = 0.01; T6: *p* = 0.04) (Table 6). Polyunsaturated FAs of the SAT were also correlated with %WL at T1 (*p* = 0.058) and at T6 (*p* = 0.048). On the other hand, at T1, saturated FAs were correlated negatively with these variables in both tissues (%WL-SAT: *p* = 0.04; %EBMIL-SAT: *p* = 0.017; %WL-VAT: *p* = 0.047; %EBMIL-VAT: *p* = 0.037) (Tables 5 and 6).

**Table 6:** Significant correlations between the groups of FAs by degree of unsaturation (saturated, monounsaturated and polyunsaturated) of SAT and the parameters: %WL, %EBMIL, and plasma concentrations of IL-6 and TNF at collection times

FA group	Baseline	1 year’ surgery	3 years’ surgery	6 years’ surgery
**Saturated FA**		**%WL** *r* = − 0.359 *p* = 0.04		
		**%EBMIL** *r* = − 0.41 *p* = 0.017		
**Polyunsaturated FA**		**%WL** *r* = 0.33 *p* = 0.058		
		**%EBMIL** *r* = 0.35 *p* = 0.04	**%EBMIL** *r* = 0.43 *p* = 0.01	**%WL** *r* = 0.35 *p* = 0.048
				**%EBMIL** *r* = 0.36 *p* = 0.042

*FA* fatty acid, *SAT* subcutaneous adipose tissue, *%WL* weight loss percentage, *%EBMIL* percentage of excess BMI that was lost., *IL-6* interleukin 6, *TNF* tumor necrosis factor alpha. Correlation test: Spearman (*p* < 0.05)

### Anthropometric variables, body composition, and tissue FA profile were predictors of weight loss 6 years after bariatric surgery

Among the various multiple linear regression models studied, the model composed of preoperative BMI, WC, HC, BF%, BMR, SAT-C18:2, and VAT-C18:0 variables was found to be statistically significant. This model explained 74.44% (*r*^*2*^ = 0.7444; *p* = 0.0039) of the changes in the percentage of weight loss at 6 years postsurgery (Table 7).

**Table 7:** Multiple linear regression model predicts the percentage of 6-year weight loss after bariatric surgery (*n* = 33)

	β Coeficient ± SE	*p*
Intercept	132.71 ± 53.56	0.035
Baseline Weight	1.39 ± 0.53	0.028
Baseline BMI	−1.63 ± 0.72	0.049
Baseline WC	−0.39 ± 0.20	0.085
Baseline HC	0.54 ± 0.15	0.006
Baseline BF%	−2.27 ± 0.88	0.029
Baseline BMR	−0.08 ± 0.03	0.015
SAT-C18:2	2.42 ± 0.53	0.001
VAT-C18:0	1.16 ± 1.16	0.005

*SE* standard error, *BMI* body mass index, *WC* waist circumference, *HC* hip circumference, *BF%* body fat percentage, *BMR* basal metabolic rate, *SAT-C18:2* C18:2 fatty acid of subcutaneous adipose tissue, *VAT-C18:0* C18:0 fatty acid of visceral adipose tissue. The model above explains 74.44% of the variations occurred in the percentage of weight loss in 6 years after bariatric surgery (*r*^*2*^ = 0.7444; *p* = 0.0039)

Based on these data, an equation was proposed to predict weight loss at 6 years and included the data related to weight, WC, and LM%. The model explains only 25.63% of the changes in the percentage of weight loss (Table 8).

**Table 8:** Equation for prediction of weight loss in 6 years after bariatric surgery (n = 33)

% WL 6 years = − 67.7 + Weight x (− 0.441) + HC × 0.812 + LM% × 0.785	

*WL* weight loss, *HC* hip circumference, *LM%* lean body mass percentage. The model above explains 25.63% of the variations occurred in the percentage of weight loss in 6 years after bariatric surgery (*r*^*2*^ = 0.2563; *p* = 0.0088)

## Discussion

In the present study, higher values of the monounsaturated FAs (MUFAs) C14:1, C16:1, and C20:1 were observed in the VAT, parallel to higher concentrations of FAs C16:0, C17:0, C20:2, C22:0, and C20:4 in the SAT. On the other hand, Pezeshkian et al. [32], in a comparison between epicardial VAT and SAT, found higher concentrations of saturated FAs (SFA) C14:0, C16:0, and C18:0 and lower concentrations of unsaturated FAs, including C16:1 (n-7), C18:1 (n- 9), C18:2 (n- 6), and C18:3 (n- 3), in the VAT.

Intra-abdominal VAT deposits drain FAs resulting from lipolysis directly into the liver via the portal circulation [4, 33]. Additionally, the severe weight loss caused by surgical procedures predisposes individuals to the development of nonalcoholic fatty liver disease (NAFLD) due to the high lipolysis rate of AT [34]. Since visceral obesity is strongly correlated with the occurrence of NAFLD [35, 36], and since SFAs are involved in at least one of the mechanisms present in NAFLD via loss of endoplasmic reticulum homeostasis, the induction of hepatocyte apoptosis and secretion of cytokines involved in the development of the disease [37, 38], it is beneficial to have a lower concentration of SFAs in VAT, as identified in the present study.

The negative effect of SFAs was observed in the positive associations between IL-6 and SAT-C14:0, SAT-C15:0 and VAT-C14:0 concentrations. However, the FA VAT C18:0 was negatively associated with TNF levels. Considering that FA C18:0 is a precursor of C18:1 and the latter is a precursor of eicosatrienoic acid (ETA; C20:3 n-9) with anti-inflammatory potential [39, 40], it is possible that the positive effect on the plasma TNF concentration was due to the conversion of C18:0 to C18:1.

On the other hand, and in agreement with the literature that reports some relationship between MUFAs and disorders related to obesity [41, 42], MUFA concentrations of SAT-C14:1, SAT/VAT-C16:1, SAT/VAT-C17:1, VAT-C18:1, and VAT-20:1 were also positively associated with plasma IL-6 concentrations. However, unlike the results reported by Kunešová et al. [9], no association was found between the content of FAs C14:1 and C16:1 with weight loss variables, and there was no predictive effect of maintenance of body weight by MUFA. Thus, the FA predictors of weight loss after 6 years of surgery were VAT-C18:0 and SAT-C18:2 (n-6), which were negatively correlated with the IL-6 concentrations and positively correlated with the parameters of weight loss and BMI.

Although the concentration of FA C18:3 in the VAT was not abundant, the results indicated an anti-inflammatory potential, since it was negatively correlated with the concentrations of IL-6 at T6. The present study’s results are consistent with those expected, since the essential FAs 18:2 (n-6) and 18:3 (n-3), in an adequate proportion, originate products with known health benefits, such as the prevention of platelet aggregation, reduction of the LDL (low density lipoprotein) and attenuation of the adverse effects of homocysteine, not to mention their anti-inflammatory and antineoplastic properties [43].

In fact, to achieve beneficial effects, it is recommended that the diet offer a ratio of 1–4:1 between FAs C18:2 (n-6) and C18:3 (n-3) [44], where the ratio of 1–2:1 is indicated as an important dietary factor for obesity prevention [45]. The ratio found in the tissues was above 20:1, while the pre-surgery diet was 10:1 (data not shown), both beyond the recommendation, which demonstrates the importance of dietary intervention for better postsurgery response.

In addition to the dietary point of view, the physiological changes caused by bariatric surgery play a role in modulating the metabolic functions in the body, represented by the change in the composition and the functional capacity of the intestinal microbiota [46]; the increase in β-cell function and insulin sensitivity in multiple organs; and the reduction of intra-abdominal adipose tissue volume, intrahepatic triacylglycerol content, systolic blood pressure and plasma triacylglycerol concentration. Moreover, the increased weight loss promoted by surgery may exert an additional benefit, represented by the progressive effect on AT gene expression related to cholesterol excretion, lipid synthesis, remodeling of the extracellular matrix, and the response to oxidative stress [47].

Therefore, further investigations of the influence of the dietary FA profile on the postsurgical response, as well as the effect of surgery on the remodeling of FA metabolism, are required.

## Conclusion

The FA profile of the AT of obese adult women may be a predictor of the ponderal and inflammatory response after 6 years of bariatric surgery, since SFAs with 14 and 15 carbons and MUFAs with 14, 15, 16, 17, 18, and 20 carbons presented a proinflammatory profile, primarily in the late postoperative period. On the other hand, the C18:3 n-3 polyunsaturated FA, although not abundant in the tissues, showed anti-inflammatory potential, whereas the polyunsaturated C18:2 n-6, in addition to being anti-inflammatory, was favorable for the loss of postsurgical weight.

Considering that the accumulation of different types of FAs in the AT is determined primarily by the lipids present in ingested foods, knowledge related to their influence on the inflammatory profile and the long-term loss of weight constitutes a perspective of dietary behavior for this type of population.

Last, the present study obtained an equation predictive of weight loss at 6 years postsurgery. Although its predictive value was low, it could be a valuable auxiliary therapeutic tool to be incorporated into treatments.

## Supplementary information

**Additional file 1: Table S1.** Characterization of the sample in the baseline. **Table S2.** Anthropometric variables, body composition and energy expenditure of women submitted to two techniques of bariatric surgery (SG or RYGB), at the baseline and at the time of 1, 3 and 6 years after bariatric surgery.

## Data Availability

Please contact the corresponding author for reasonable data requests.

## Abbreviations

%EBMIL: Percentage of excess BMI that was lost
%WL: Weight loss percentage
BF%: Body fat percentage
BIOAGRO: Institute of Biotechnology Applied to Agriculture
BMI: Body mass index
BMR: Basal metabolic rate
ETA: Eicosatrienoic acid
FA: Fatty acid
FAME: Fatty acid methyl esters
HC: Hip circumference
IL-6: Interleukin 6
LDL: Low density lipoprotein
LM%: Lean body mass percentage
LTB4: Leukotriene B4
MUFA: Monounsaturated fatty acid
NAFLD: Nonalcoholic fatty liver disease
RYGB: Roux-en-Y bypass
SAT: Subcutaneous adipose tissue
SG: Sleeve gastrectomy
TNF: Tumour necrosis factor alpha
UFES: Federal University of Espírito Santo
UFV: Federal University of Viçosa
VAT: Visceral adipose tissue
WC: Waist circumference
WHR: Waist hip ratio
WL: Weight loss
