# Supplementary material for: The fatty acid profile of adipose tissue as a predictor of the ponderal and inflammatory response in adult women six years after bariatric surgery
Source: Lipids Health Dis. 2020 Mar 16;19:45. doi: 10.1186/s12944-020-01229-3 (PMC7077013; doi:10.1186/s12944-020-01229-3)
Supplement: Supplementary file 1 — Additional file 1: Table S1. Characterization of the sample in the baseline. Table S2. Anthropometric variables, body composition and energy expenditure of women submitted to two techniques of bariatric surgery (SG or RYGB), at the baseline and at the time of 1, 3 and 6 years after bariatric surgery. [file 12944_2020_1229_MOESM1_ESM.docx]

**Supplemental** **table 1** Characterization of the sample in the baseline.

| **Variable** | **SG + RYGB** | **SG** | **RYGB** |
| --- | --- | --- | --- |
| **Age (years)** | 41.54 + 8.37 | 42.42 + 8.73 | 40.36 + 8.01 |
| **Weight (Kg)** | 108.00 (103.90 – 113.00) | 107.10 (103.85 – 111.85) | 112.40 (104.63 – 120.90) |
| **Height (m)** | 1.61 + 0.06 | 1.60 + 0.05 | 1.63 + 0.07 |
| **Initial BMI (Kg/m^2^)** | 42.20 (41.50 – 43.57) | 42.20 (41.39 – 43.32) | 42.60 (41.55 – 44.19) |
| **WC (cm)** | 118.61 + 7.81 | 117.60 + 6.50 | 119.96 + 9.39 |
| **BF%** | 49.51 + 2.86 | 49.05 + 3.02 | 50.13 + 2.61 |
| **LM%** | 50.20 (48.40 – 52.00) | 51.50 (49.70 – 52.85) | 49.40 (48.25 – 50.48) |
| **BMR (Kcal)** | 1713.76 + 151.98 | 1681.16 + 134.92 | 1758.00 + 167.30 |

Values presented by mean + standard deviation or median (interquartile range [IQR]). Shapiro-Wilk followed by t test or Mann-Whitney. SG = sleeve gastrectomy (n = 19); RYGB = Roux-en-Y bypass (n = 14); BMI = body mass index; WC = waist circumference; BF% = body fat percentage; LM% = lean body mass percentage; BMR = basal metabolic rate.

**Supplemental table 2** Anthropometric variables, body composition and energy expenditure of women submitted to two techniques of bariatric surgery (SG or RYGB), at the baseline and at the time of 1, 3 and 6 years after bariatric surgery.

| **Variable** | **Baseline** | | | **1 year’ surgery** | | | **3 years’ surgery** | | | **6 years’ surgery** | | |
| --- | --- | --- | --- | --- | --- | --- | --- | --- | --- | --- | --- | --- |
|  | **Median (mean + standard deviation)** | | | **Median (mean + standard deviation)** | | | **Median (mean + standard deviation)** | | | **Median (mean + standard deviation)** | | |
|  | **SG + RYGB** | **SG** | **RYGB** | **SG + RYGB** | **SG** | **RYGB** | **SG + RYGB** | **SG** | **RYGB** | **SG + RYGB** | **SG** | **RYGB** |
| **Weight (kg)** | 108.0 a  (110.2+9.6) | 107.1a  (107.7+6.6) | 112.4a  (113.7+12.1) | 72.0c  (71.9 + 9.2) | 71.4c  (70.9 + 9.9) | 73.7c  (74.2+8.1) | 71.4c  (72.6+10.3) | 70.3c  (71.7+11.5) | 72.1c  (73.8+8.8) | 74.5b  (78.4+13.6) | 73.6b  (76.1+11.5) | 79.6b  (81.6+16.0) |
| **BMI (Kg/m²)** | 42.2a  (42.3+2.3) | 42.2ª  (41.9+2.71) | 42.6a  (42.8+1.5) | 27.3c  (27.5+2.4) | 27.4c  (27.2+2.4) | 27.1c  (28.0+2.5) | 27.5c  (27.7+3.1) | 27.4c  (27.7+3.3) | 27.9c  (27.7+3.1) | 28.9b  (30.0+4.2) | 28.5b  (29.7+3.1) | 29.3b  (30.5+5.4) |
| **WC**  **(cm)** | 118.5a  (118.6+7.8) | 118.0a  (117.6+6.5) | 121.7a  (120.0+9.4) | 90.0b  (90.8+7.4) | 88.0bc  (90.3+6.8) | 90.0b  (91.4+8.4) | 81.5c  (85.6+9.9) | 81.5c  (86.0+10.3) | 83.7c  (85.0+9.6) | 92.8b  (92.2+11.7) | 96.0b  (92.7+10.2) | 92.1b  (91.5+13.8) |
| **HC**  **(cm)** | 129.0a  (129.6+8.4) | 129.0a  (129.2+5.3) | 130.25a  (130.1+11.6) | 103.5c  (102.7+6.8) | 101.5b  (100.2+7.1) | 107.1c  (106.1+4.5) | 105.0c  (106.0+6.8) | 103.8d  (105.7+7.0) | 105.7c  (106.4+6.2) | 110.6b  (112.1+9.2) | 109.7c  (110.7+8.0) | 113.1b  (114.0+10.6) |
| **WHR (cm)** | 0.9a  (0.9+0.1) | 0.9a  (0.9+0.1) | 0.9a  (0.9+0.1) | 0.9a  (0.9+0.1) | 0.9a  (0.9+0.0) | 0.8a  (0.9+0.1) | 0.8b  (0.8+0.1) | 0.8b  (0.8+0.1) | 0.8b  (0.8+0.1) | 0.8b  (0.8+0.1) | 0.8b  (0.8+0.1) | 0.8b  (0.8+0.1) |
| **BF%** | 49.8a  (49.5+2.9) | 48.5ª  (49.0+3.0) | 50.6a  (50.1+2.6) | 32.2c  (32.7+6.4) | 32.0d  (31.3+5.2) | 33.3d  (34.0+7.8) | 35.0c  (34.6+4.9) | 35.0c  (35.5+4.9) | 34.7c  (33.4+5.0) | 38.7b  (38.3+6.4) | 38.7b  (38.7+5.2) | 38.1b  (37.9+7.9) |
| **LM%** | 50.2c  (51.0+4.5) | 51.5c  (51.0+3.0) | 49.4c  (50.9+6.2) | 67.8a  (67.2+6.6) | 68.0a  (68.3+5.2) | 66.7ab  (65.9+8.1) | 65.0a  (65.4+5.0) | 65.0b  (64.5+4.9) | 65.3a  (66.6+5.0) | 61.3b  (61.6+6.4) | 61.3b  (61.3+5.2) | 61.9b  (62.1+7.9) |
| **BMR**  **(Kcal)** | 1707.0a (1713.8+152.0) | 1668.0a  (1681.2+134.9) | 1728.0a  (1758.0+167.3) | 1541.0b (1581.6+225.0) | 1474.0Bb  (1505.8+124.6) | 1632.0Aab (1684.6+288.5) | 1541.0bc  (1514.4+138.4) | 1466.0b (1477.7+141.3) | 1578.0b  (1564.3+121.8) | 1493.0c  (1459.2+290.5) | 1471.0Bb  (1388.0+350.5) | 1544.0Ab  (1555.8+142.4) |

Values presented by median (mean + standard deviation). Lowercase letters indicate the difference (*p* < 0.05) among the groups at each collection time (Shapiro-Wilk followed by t test or Mann-Whitney). Upper case letters indicate difference (*p* < 0.05) among collection times for each group (Repeated measures ANOVA, followed by the Tukey-Kramer test for parametric data; or Friedman test followed by the Dunn test for non-parametric data). SG = sleeve gastrectomy (n = 19); RYGB = Roux-en-Y bypass (n = 14); BMI = body mass index; HC = hip circumference; WC = waist circumference; WHR = waist hip ratio; BF% = body fat percentage; LM% = lean mass percentage; BMR = basal metabolic rate.
